# Comparison Between Magnetic Resonance Imaging and Computed Tomography in the Detection and Volumetric Assessment of Lung Nodules: A Prospective Study

**DOI:** 10.3389/fmed.2022.858731

**Published:** 2022-04-28

**Authors:** Emeline Darçot, Mario Jreige, David C. Rotzinger, Stacey Gidoin Tuyet Van, Alessio Casutt, Jean Delacoste, Julien Simons, Olivier Long, Flore Buela, Jean-Baptiste Ledoux, John O. Prior, Alban Lovis, Catherine Beigelman-Aubry

**Affiliations:** ^1^Department of Diagnostic and Interventional Radiology, Lausanne University Hospital (CHUV), Lausanne, Switzerland; ^2^Faculty of Biology and Medicine, University of Lausanne (UNIL), Lausanne, Switzerland; ^3^Department of Nuclear Medicine and Molecular Imaging, Lausanne University Hospital (CHUV), Lausanne, Switzerland; ^4^Department of Pulmonology, Lausanne University Hospital (CHUV) and University of Lausanne (UNIL), Lausanne, Switzerland; ^5^Department of Physiotherapy, Lausanne University Hospital (CHUV) and University of Lausanne, Lausanne, Switzerland; ^6^Center for Biomedical Imaging (CIBM), Lausanne, Switzerland

**Keywords:** lung MRI, MR nodule detection, CT nodule detection, nodule volume assessment, high-frequency noninvasive ventilation

## Abstract

**Rationale and Objectives:**

Computed tomography (CT) lung nodule assessment is routinely performed and appears very promising for lung cancer screening. However, the radiation exposure through time remains a concern. With the overall goal of an optimal management of indeterminate lung nodules, the objective of this prospective study was therefore to evaluate the potential of optimized ultra-short echo time (UTE) MRI for lung nodule detection and volumetric assessment.

**Materials and Methods:**

Eight (54.9 ± 13.2 years) patients with at least 1 non-calcified nodule ≥4 mm were included. UTE under high-frequency non-invasive ventilation (UTE-HF-NIV) and in free-breathing at tidal volume (UTE-FB) were investigated along with volumetric interpolated breath-hold examination at full inspiration (VIBE-BH). Three experienced readers assessed the detection rate of nodules ≥4 mm and ≥6 mm, and reported their location, 2D-measurements and solid/subsolid nature. Volumes were measured by two experienced readers. Subsequently, two readers assessed the detection and volume measurements of lung nodules ≥4mm in gold-standard CT images with soft and lung kernel reconstructions. Volumetry was performed with lesion management software (Carestream, Rochester, New York, USA).

**Results:**

UTE-HF-NIV provided the highest detection rate for nodules ≥4 mm (*n* = 66) and ≥6 mm (*n* = 32) (35 and 50%, respectively). No dependencies were found between nodule detection and their location in the lung with UTE-HF-NIV (*p* > 0.4), such a dependency was observed for two readers with VIBE-BH (*p* = 0.002 and 0.03). Dependencies between the nodule's detection and their size were noticed among readers and techniques (*p* < 0.02). When comparing nodule volume measurements, an excellent concordance was observed between CT and UTE-HF-NIV, with an overestimation of 13.2% by UTE-HF-NIV, <25%-threshold used for nodule's growth, conversely to VIBE-BH that overestimated the nodule volume by 28.8%.

**Conclusion:**

UTE-HF-NIV is not ready to replace low-dose CT for lung nodule detection, but could be used for follow-up studies, alternating with CT, based on its volumetric accuracy.

## Introduction

Lung nodule assessment is routinely performed with CT. It may concern patients with incidental findings or oncologic patients, and appears very promising for lung cancer screening (1–3). Nevertheless, even with low-dose CT questions remain regarding the potential health hazard due to radiation exposure with time (4). Meanwhile, the development of new MR sequences (5) has highlighted the potential of MRI as a radiation-free imaging modality of great interest for lung nodule assessment (6–8). Moreover, given that acquisition at full lung volume is of major importance for optimized management of indeterminate pulmonary nodules, a study has been investigating the combination of a high-frequency noninvasive ventilation (HF-NIV) technique that allows an apnea-like respiratory stabilization of several minutes at full lung volume with lung MRI. The higher image quality provided by MR images acquired under HF-NIV vs. without has been recently demonstrated in healthy volunteers (9). Several MR sequences such as UTE Spiral VIBE (volumetric interpolated breath-hold examination) without and with correction and 3D radial UTE have also been investigated under HF-NIV and the later was the sequence that would provide the highest image quality scores (9, 10).

Based on these initial results, the goal of this prospective study was to evaluate the capability of lung MRI in terms of sequence and acquired lung volume for lung nodule detection and volume measurement in comparison with the gold standard CT modality. The long-term perspective of this study was to determine whether MRI is well-suited for lung nodule assessment, this including lung cancer screening, as considered by several authors (11, 12).

## Materials and Methods

Permission from the State of Vaud Ethic Committee (CER-VD, 2018-00438) was obtained for this prospective study. The recruitment of participants was conducted consecutively from 31 January 2019 to 16 March 2020. All patients provided written informed consent prior to enrollment. Please note that one patient of this study participated previously to two other published studies (13, 14). During these studies, where he was enrolled as volunteer, we discovered incidental nodules that required follow-up by low dose CT. He therefore was enrolled as a patient in this study while coming for his follow-up exam.

### Inclusion and Exclusion Criteria

Two inclusion criteria were defined: patients should be ≥18 years old and with at least 1 non-calcified pulmonary nodule with a long axis ≥4 mm. The exclusion criteria are gathered in Table 1.

**Table 1:** Exclusion criteria.

Previous or current disorders that might interfere with performance or safety of study procedures
Age <18 years
Pregnant or breastfeeding women
Any contraindication to MRI (pacemakers, neurostimulators, some implantable devices, some metallic implants, claustrophobia)
Adults with mental incapacities
Inability to follow the procedures of the study e.g., due to language problems, psychological disorders, dementia, etc. of the participant
COPD or asthma with severe obstruction: severe obstructive patients (FEV1 <50% of predicted value), Hypoxemia (SaO_2_ < 94% AA), history or physical signs of right heart failure.
History or physical signs of right or left cardiac failure
History or physical signs of pulmonary hypertension
History or physical signs of active coronary artery disease
Pulmonary graft
Immunocompromised patients
Known or suspected non-compliance with appointments, alcoholism, drug addiction or alike
Enrolment of the investigator, his/her family members, employees and other dependent persons

*Respiratory and cardiac conditions listed in this table are defined according to the use of the HF-NIV*.

*COPD, Chronic Obstructive Pulmonary Disease; FEV, Forced Expiratory Volume; SaO_2_, Oxygen Saturation Rate*.

**Figure 1:**
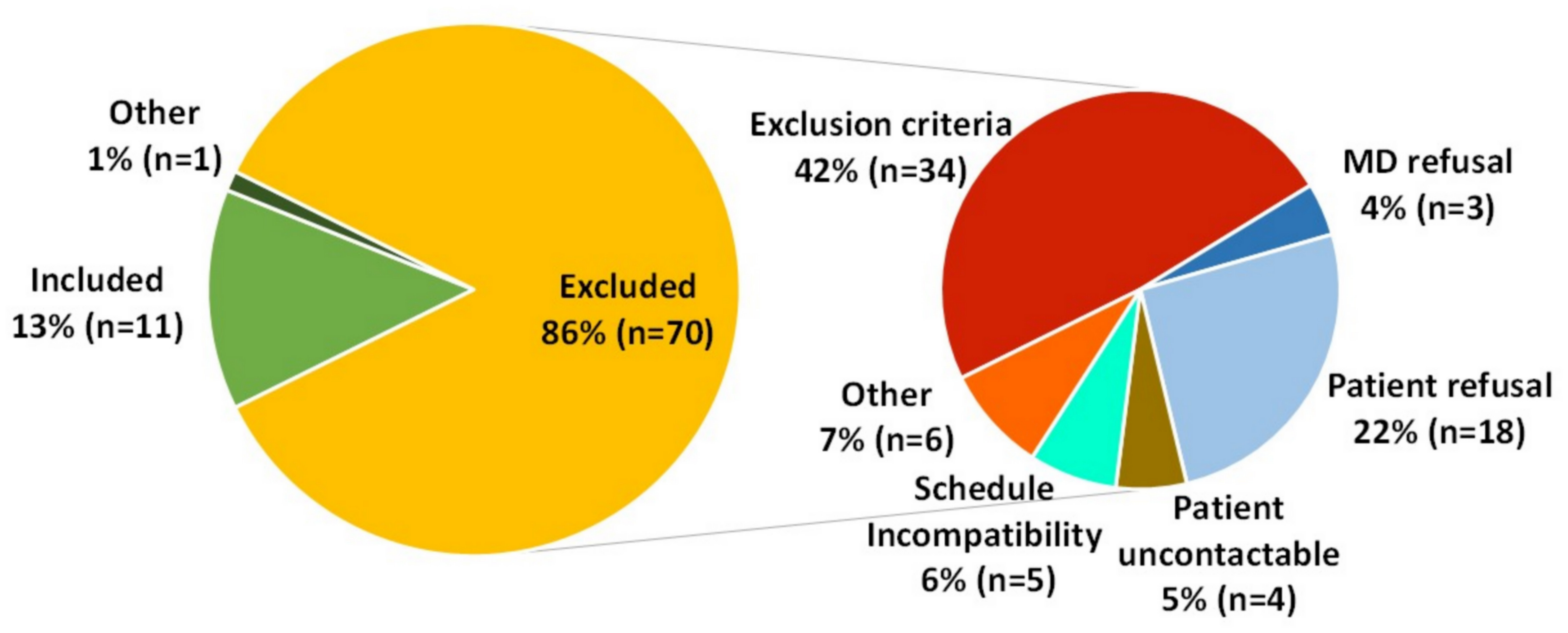
Details of the patient inclusion and exclusion during the screening.

Eighty-two patients were identified as presenting the inclusion criteria. Seventy (85%) patients were not eligible for various causes (Figure 1), 1 patient agreed to participate but could not due to personal reasons.

### HF-NIV Technique

The HF-NIV was performed on patients with a Monsoon III ventilator (Acutronic Medical Systems, Hirzel, Switzerland) and a non-invasive patient interface (Phasitron, Percussionnaire, Idaho, USA) (14), according to a set-up described in Delacoste et al. (9). The oxygen delivery has been set to 100% at the monitor. In practice, given the ingress of ambient air through the inspiratory port of the Phasitron ®, the fraction of inspired oxygen (FiO_2_) was ~50% (10).

Before the MRI exam, to assess the tolerance to the HF-NIV and to ensure a respiratory stabilization period ≥6 min at a respiratory rate of 250/min in agreements with Ogna et al. (15), each patient was trained by a physiotherapist during a screening session. The following physiological parameters were also monitored by a pulmonologist with a Digital Monitoring System (SenTec, Therwil, Switzerland): continuous transcutaneous capnography (TcCO_2_), oxygen saturation (SpO_2_) and cardiac frequency. Arterial pressure was also monitored. Eligible patients underwent the MRI exam less than a month after the CT exam and due to MR constraints only SpO_2_ and cardiac frequency were then controlled.

### Data Acquisition and Reconstruction

All data acquisitions were performed on a 3T clinical MR scanner (MAGNETOM PrismaFit, software version VE11D, Siemens Healthcare, Erlangen, Germany). All patients were positioned feet first, supine, with arms at their side. A 16-channel body array combined to 32-channel spine coils were used. Shimming was performed ahead of the acquisitions to correct for the magnetic field inhomogeneities.

A prototype double echo Ultra-Short Echo time (UTE) sequence (16, 17) with spiral phyllotaxis trajectory (18) was acquired without any triggering under HF-NIV (UTE-HF-NIV) and in free-breathing (UTE-FB), with the following parameters: echo times TE_1_/TE_2_ = 0.08 ms/2.86 ms, repetition time TR = 5.9 ms, readout bandwidths BWr_1_/ BWr_2_ = 305 Hz/Px/610 Hz/Px, radio-frequency excitation angle = 5°, field of view (FOV) = (250 mm)^3^, voxel size = 1 mm^3^, 1,220 segments of 50 readouts each and an acquisition time of 6 min. The UTE-FB at tidal volume was always acquired before the HF-NIV acquisition at full inspiration to avoid any change in breathing related to the potential recovery after the HF-NIV procedure.

The reconstruction was performed off-line with Matlab (Version R2018b, The Mathworks, Natick, Massachusetts, USA) and a non-uniform fast Fourier transform. For FB data, a compressed sensing algorithm that exploited the sparsity over the respiratory dimension was used to obtain respiratory-motion-resolved images. Only end-expiration images were kept for analysis. If the patient happened to breathe before the end of the HF-NIV acquisition, the motion-corrupted data were removed from the raw data set prior to the off-line reconstruction.

A VIBE sequence with asymmetric sampling in the readout dimension was also acquired during an unassisted breath-hold (BH) on inspiration (VIBE-BH). The acquisition parameters were as follows: TE = 0.94 ms, TR = 4.0 ms, BWr = 500 Hz/Px, radio-frequency excitation angle = 5°, FOV = 400 × 262.4 mm^2^, voxel size = 1 × 1 × 3 mm^3^, slice partial Fourier = 7/8, orientation = transversal, CAIPIRINHA acceleration factor = 4, T_acq_ = 20 s. Images were directly reconstructed at the MR scanner.

The gold standard was a CT exam based on the ALARA principle and performed on a Revolution or Discovery scanner (General Electric, Milwaukee, USA, *n* = 7) or SOMATOM Force scanner (Siemens Healthcare, Erlangen, Germany, *n* = 1) (19). A low-dose and without intravenous contrast chest CT with a CTDI below 3 mGy [0.5–1.99], corresponding to a dose below 3 mSv, as recommended by the Fleichner Society and ACR recommendations was applied in all but one patient (20). The latter had a CT in the setting of oncological context with IV contrast administration and normal radiation dose. The mean of low dose CTs corresponded to an effective dose of 0.61 mSv. The normal dose CT performed in the setting of oncological follow-up corresponded to an effective dose of 3.32 mSv. Thin slices, with a slice thickness 1.25 mm, FOV 320 mm with in plane voxel size 0.625 mm, were used with soft and lung kernel reconstructions.

### Nodule Detection and Morphological Evaluation

MR nodule's detection analysis was carried out by three experienced chest radiologists (reader 1 (R1) >20 years, R2, 8 years and R3, 4 years of experience in thoracic imaging) on anonymized and randomly sorted images. MR volume assessment was performed by the most experienced reader of the three (R1). Anonymized gold standard CT images were analyzed by two readers (R1 and R4, 5 years of experience in thoracic imaging). To avoid any bias, the common reader (R1) first performed the nodule's detection on MR images and after a 2 weeks period performed the reading of gold standard CT images.

The nodule detection was determined according to the following visual score: absent = 1; probably absent = 2; uncertain = 3; probably present = 4; present = 5. The true positive, false negative and false positive nodules were reported for each technique and each reader. The detection rate of each technique was assessed for nodules ≥4 mm and ≥6 mm by computing the fraction of nodules with a visual score >3, i.e., considered as present, in MR images and confirmed on CT. The two MR techniques that provided the highest detection rate were used for further analyses.

Evaluated morphological criteria included the long axis (l_axis_), the mean diameter (d_mean_, average of the long and short axes), the solid/subsolid texture and the intrapulmonary lymph node nature or not of solid nodules. The distance to the pleura (d_pleura_) was assessed and nodules were divided within superior, middle or inferior areas for nodules in segments 1 to 3, 4 to 6, and 7 to 10, respectively, according to the Boyden classification.

The detection's dependency on the nodules' d_pleura_ and superior-middle-inferior location was investigated as well as on the l_axis_, d_mean_ and measured volume in soft kernel images (V_soft_).

### 2D and 3D Measurements

The 2D-l_axis_ measurements were compared between the CT images and images of the two MR techniques with the highest detection rate of MR detected nodules. 3D measurements were performed on CT, and on maximum five randomly selected nodules per patient among all included nodules for MRI, this to minimize bias. When a selected nodule was not detected with one of the two investigated MR techniques, another nodule was selected, when available.

The nodules' volume was measured with the lesion management software (Carestream, Rochester, New York, USA), both on lung and soft kernel CT images by one reader, and soft kernel CT images only by a second reader. The volumetric measurements performed by the more experienced reader in soft kernel images were considered the reference value. Semi-automatic contouring was used by default for CT and manual drawing was used for MR and when necessary for CT. 3D volumetric measurements were compared between lung and soft kernel CT images and MR images.

### Statistical Analyses

The formula of Beam et al. was used to determine the minimal sample size of nodules required to demonstrate an improvement in sensitivity of 90% with a power of 80% (21). A two-sided Fisher's exact test was applied to investigate the dependency between the nodules' detection and their location in superior, mid, or inferior lung areas. To investigate the nodules' detection dependency on d_pleura_, l_axis_, d_mean_, and V_soft_, Wilcoxon signed-rank tests were performed. Results were considered significant when *p* < 0.05. For the volume assessment analyses, Lin's concordance correlation analyses (22), Bland-Altman agreement plots and paired Wilcoxon signed-rank tests were performed. Inter- and intra-reader variabilities were addressed with Bland-Altman plots.

## Results

### Patients Cohort

Over the 11/82 (13%) included patients, 8 patients (age 54.9 ± 13.2 years, weight 72.8 ± 7.9 kg, 2 women) successfully completed the study (MR-HF-NIV stabilization period: 5.63 min, range [4–6]) without any adverse events. Three patients did not complete it for the following reasons: 1 could not achieve the MR-HF-NIV sequence until the end and 2 canceled the MRI session for reasons unrelated to the study.

### Nodule's Characteristics

Based on the CT gold standard analysis, 66 nodules ≥4 mm were included (mean size 6 ± 3 mm, range 4–23 mm; 22 of 4 mm, 12 of 5 mm, 11 of 6 mm and 21 > 6 mm of diameter), thus above the determined minimal sample size of 28. Three nodules were classified as subsolid and 63 as solid, among which 12 were classified as lymph nodes. While none of the subsolid nodules were identified as such in MR images, one reader identified properly 4 lymph nodes out of 12 in the VIBE-BH images and 1 lymph node out of 12 in the UTE-HF-NIV images.

### Detection Rate

**Figure 2:**
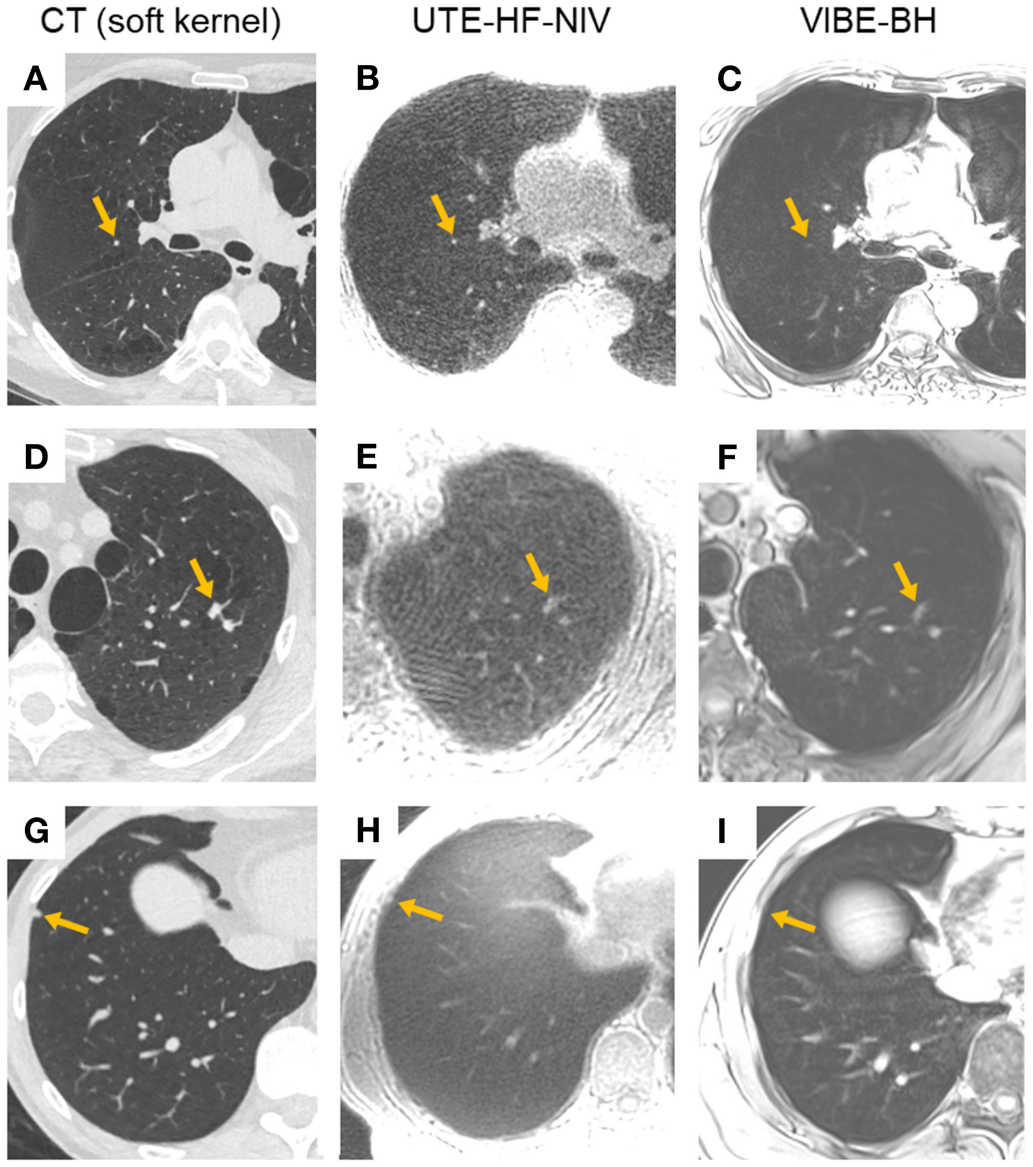
Examples of nodules not reported in MR analyses. **(A–C)** CT, UTE-HF-NIV, and VIBE-BH images of the right upper lobe showing a juxta-fissural nodule of 4-mm long axis (orange arrow), respectively. While visible in UTE-HF-NIV image **(B)**, its long axis was measured <4 mm, hence not reported. The nodule was scarcely seen in **(C)**. **(D–F)** CT, UTE-HF-NIV, and VIBE-BH images of the left upper lobe with 2 nodules of 9 mm (orange arrow) and 6-mm-long axis, respectively. Due to a loss in morphological characteristics in both MR images, and although the neighboring nodule was easily recognized, the largest finding was considered as a parenchymal band instead of a nodule for two of the three readers, and as a nodule for the third reader. **(G–I)**. CT, UTE-HF-NIV, and VIBE-BH images of the right lower lobe with a nodule of 5-mm long axis (orange arrow), respectively. The nodule was barely seen on UTE-HF-NIV image **(H)** and reported as absent in both MR images.

UTE-HF-NIV images provided the highest detection rate for two of the three readers and for the two evaluated sizes of nodule ≥ 4 mm (*n* = 66) and ≥ 6 mm (*n* = 32) (35 and 50%, respectively), while UTE-FB provided the lowest detection rate for all readers (Table 2). False negatives were related either to the invisibility of the nodule, to a l_axis_ measurement <4 mm on MR images while ≥ 4 mm in CT images, or because findings were attributed to subpleural abnormalities or parenchymal bands (Figure 2). Depending on the reader, UTE-HF-NIV image analysis resulted in 4 to 9 false positives (false positive rate 6–14%) while VIBE-BH images analyses resulted in 4 to 6 false positives (false positive rate 6–9%).

**Table 2:** Detection rate with MR modality and the three investigated methods.

	**Detection rate** **Nodules' long axis ≥4 mm (*****n*** **= 66) [%]**			**Detection rate** **Nodules' long axis ≥6 mm (*****n*** **= 32) [%]**		
	**R1**	**R2**	**R3**	**R1**	**R2**	**R3**
VIBE-BH	33	29	26	47	41	44
UTE-HF-NIV	30	35	32	44	47	50
UTE-FB	12	20	15	22	34	28

*CT imaging was used as gold standard*.

*R, reader; VIBE-BH, Volumetric interpolated breath-hold examination sequence during an unassisted breath-hold; UTE-HF-NIV, Ultra-Short Echo time sequence under High-Frequency Noninvasive Ventilation; UTE-FB, Ultra-Short Echo time sequence in free-breathing*.

**Figure 3:**
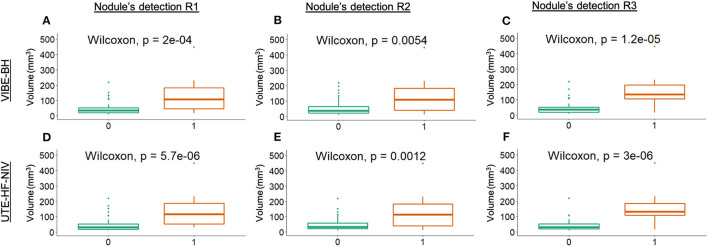
Dependency between nodule's detection and the nodule's soft kernel volume measurement. Boxplots of the dependency investigated in the VIBE-BH images **(A–C)**, and in the UTE-HF-NIV images **(D–F)** by the three readers, respectively. The nodule detection was represented by a binary classification: 0 for undetected nodules (visual score <4), 1 for detected nodules (visual score >3). Two outliers of 1,068 and 2,572 mm^3^ from the detected group have been omitted from the graphs to provide an adapted scale.

With the UTE-HF-NIV, no dependencies were observed between the nodule's detection and its superior-middle-inferior location in the lung (*p* = 0.45, 0.55 and 1 for the three readers). One reader confirmed these results for the VIBE-BH, conversely to the two other readers who reported significant differences between superior, middle or inferior location of the nodules. Except for two readers with the UTE-HF-NIV (*p* = 0.002, *p* = 0.04), no dependencies were found between the nodule's detection and its d_pleura_ (*p* = 0.8, 0.5 and 0.8 for VIBE-BH, *p* = 0.05 for UTE-HF-NIV). For all readers and the two investigated techniques, there was a significant dependency between the MR nodule's detection and the nodules' measured l_axis_, d_mean_ and V_soft_ (*p* < 0.02, Figure 3).

### Nodule Size Assessment

**Figure 4:**
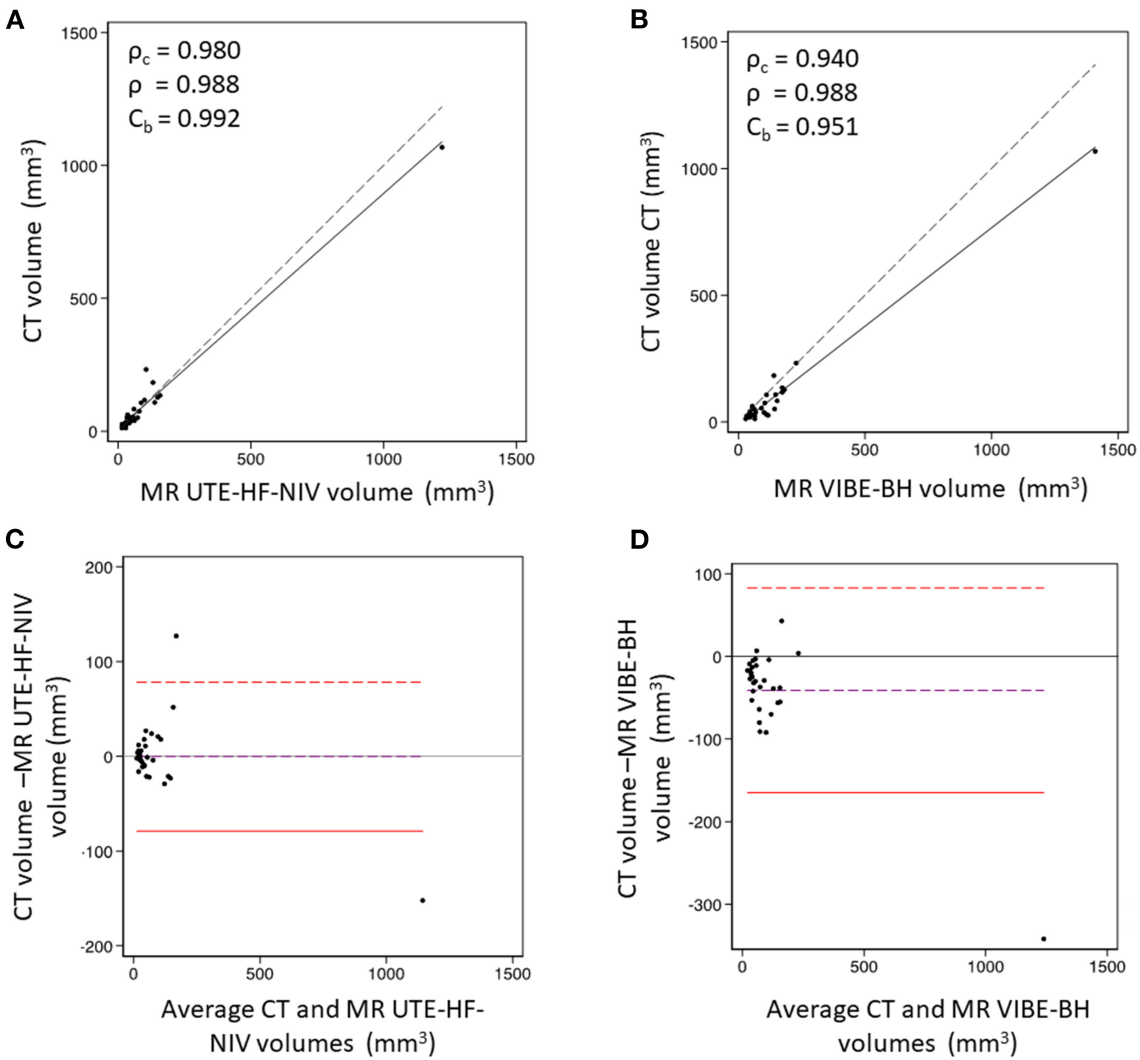
Nodule volume measurement comparison between CT (soft kernel) and MR images. **(A)** Lin's concordance analysis between CT and UTE-HFNIV. The graph shows an excellent concordance that corresponds to an overestimation of 13.2% of nodule volumes by UTE-HF-NIV compared to CT soft kernel. **(B)** Lin's concordance analysis between CT and VIBE-BH. VIBE-BH overestimated the lung nodule volume by 28.8%, with a significantly lower concordance as compared to UTE-HF-NIV (*p* < 0.05). Dashed line is the line of equality. **(C)** Bland-Altman plot comparing CT soft kernel and UTE-HF-NIV MRI based volume nodule measurements. A bias of −0.16 mm^3^ was observed (limits of agreements −79 to 78 mm^3^). **(D)** Bland-Altman plot comparing CT soft kernel and VIBE-BH MRI based volume nodule measurements. A bias of −41 mm^3^ was observed, with limits of agreements (−165 to 83 mm^3^). These were based on a random selection of nodules when the number of nodules per patient exceeded 5. Dashed purple line is the observed average agreement. Dashed red lines are the 95% limits of agreement.

Given that UTE-HF-NIV and VIBE-BH had the highest detection rate among all 3 readers, the nodule size assessment was carried out with these two MR techniques. The 2D-l_axis_ measurements comparison resulted in a bias of 1.6 mm (*p* = 0.002, *n* = 22 nodules) between CT and VIBE-BH and 1.7 mm (*p* = 0.003, *n* = 20 nodules) between CT and UTE-HF-NIV. MR nodule volume assessment was performed on 31 nodules (mean V_soft_ = 94 mm^3^, range 12–1,068 mm^3^) in UTE-HF-NIV and VIBE-BH images. Regarding CT, no significant differences were observed when the same reader measured the nodule volumes in either soft or lung kernel images (*p* = 0.2) nor when measurements were compared between the two readers in soft kernel images (*p* = 0.3). When comparing nodule volume measurements between CT soft kernel and UTE-HF-NIV, Lin's concordance ρ_c_ and Pearson's correlation ρ values were fairly similar, suggesting no systematic bias with an excellent concordance for measured nodule volume (ρ_c_ = 0.980 [95%CI 0.969–0.991], ρ = 0.988, C_b_ = 0.992), corresponding to an overestimation of 13.2% of nodule volumes by UTE-HF-NIV compared to CT soft kernel (Figures 4A,B). On the other hand, VIBE-BH overestimated the nodule volume by 28.8%, with a significantly lower concordance (ρ_c_ = 0.940 [95%CI 0.913–0.968], ρ = 0.988, C_b_ = 0.951) as compared to UTE-HF-NIV (*p* < 0.05). Bland-Altman plots demonstrated almost no bias in nodule volume measurements between CT and UTE-HF-NIV as compared to VIBE-BH (bias = −0.16 mm^3^ and −41 mm^3^, respectively, Figures 4C,D).

## Discussion

Lung nodule management is firstly based on CT with a baseline assessment of its size, location, as well as its solid/subsolid texture, margins, and internal content (23). Although there are well-known CT criteria suggesting the benign or malignant nature of a nodule, most nodules remain indeterminate. The current management of this indeterminate category is heavily based on volumetric analysis, due to the lower sensitivity of the diameter measurement to growth (24). According to major guidelines, the category of solid indeterminate nodules from 30–100 mm^3^ or 4–6 mm to <200–300 mm^3^ or <8–10 mm requires at least a low-dose CT exam 3 months after detection (25, 26) to evaluate their volume doubling time, which helps to establish the probability of malignancy.

Nevertheless, and despite significant efforts on dose reduction (27), the potential danger of radiation exposure (4) remains a major concern. The goal of our study was therefore to evaluate the MRI capability to detect nodules ≥4 mm and ≥6 mm in optimized conditions, and to assess their volume. A minimal nodule size of 4 mm was chosen to cover various clinical situations considered in major guidelines (23, 25, 26).

Despite previous results reporting a high lung nodule detection rate with MR (11, 28–30), disappointing results were observed in our study with only 35% of detected nodules ≥ 4 mm and 50% ≥6 mm with the best technique, UTE-HF-NIV. The improved detection rates observed with the UTE-HF-NIV and VIBE-BH compared to UTE-FB that detected at best only 20% of nodules can be explained by the lung volume used, tidal volume for UTE-FB and deep inspiration for the two other techniques. This reinforces the need for MR sequences to be performed in deep inspiration, when abnormalities located at the lung bases are less susceptible to be under-recognized.

While there was a predominance of nodules in the superior part of the lung compared to the mid or inferior parts, no bias was observed in the nodule detection with the UTE-HF-NIV, as opposed to the VIBE-BH. On the contrary, the nodule detection dependence to d_pleura_ observed with UTE-HF-NIV illustrates a potential lack of sensitivity for peripheral nodules (10).

In all cases, the detectability scores of 50% for nodules ≥6 mm do not allow to yet consider MR as an imaging modality for lung nodule detection. In addition, taking into account the small difference in terms of detection rate between UTE-HF-NIV and VIBE-BH, and given the arduousness of the former technique, which requires specific skills in terms of pulmonology and physiology, VIBE-BH is worth investigating.

Regarding 3-D volume measurements, we found an excellent concordance between CT and MRI using UTE-HF-NIV, with a nodule volume overestimation of 13%, below the generally-agreed threshold of 25% used for growth and prediction of a nodule's malignancy risk (25, 31). This is in agreement with the very good concordance demonstrated between MR UTE-HF-NIV and CT in phantoms and one preliminary human dataset (13). The overestimation was significantly higher with the VIBE-BH (29%, *p* < 0.05) and might be explained by the 3-mm thick slices of the sequence (32, 33). Therefore, although the detection rate was lower with MRI than with CT, our results suggest that a nodule visible on UTE-HF-NIV images could be appropriately followed up by the same technique and that CT and MR volumetries can be derived from each other. While it is known from literature that nodule volume may be influenced by various respiratory phases, only images at full inspiration were used for volume assessment in this study. In this way, the investigation of the influence of different respiratory states on the nodule's volume were considered beyond the scope of this study (34, 35).

The dependency of the nodule detection with its size also sheds light on the need for high-resolution and high-signal-ratio sequences, which might be brought in a near future by the high-performance of low-field-strength MRI, which offers advantages in high-susceptibility regions such as the lungs (36).

Several limitations of our study might partially explain the observed low MRI detection rates as compared to CT. First, the 3-D radial UTE sequence was chosen to be used with HF-NIV based on the image quality investigation carried out by Darçot et al. (10), which appears not automatically to be the best sequence for nodule detection. Nevertheless, respiratory-gated UTE sequences led to a few false negatives in the literature (37, 38). Our study's false negatives were firstly related to nodules measured <4 mm with MR, while measured ≥ 4 mm with CT. A systematic underestimation of l_axis_ was indeed observed, both with VIBE-BH and UTE-HF-NIV. This is in agreement with the findings of Wielputz et al. (38), and can be partially explained by the MRI tendency to smooth the margins of structures. This systematic underestimation might therefore explained the 15 to 20 out of 22 nodules of 4 mm non recognized depending on the reader, as well as the 9 to 12 out of 12 nodules of 5 mm non recognized. This represents 32 of the 66 nodules, i.e., around 50% of included nodules. Interestingly, no bias was observed in nodule volume's measurements probably due to the higher number of voxels used for volume calculation, which might have smoothed out a potential bias generated by the soft margins and partial volume effect in MR. The average size of nodules might also explain some difference that can be found with literature: our population had a majority of small nodules, with only 21 nodules with a size >6 mm (32%), compared to the literature (39). In addition, a challenge to overcome was the numerous findings in the upper sub-pleural area that may be difficult to classify as nodules or not. Next, residual minimal motion related to the ventilation technique was responsible of tiny artifacts competing with VIBE sequences in short apnea. In all cases, our results should be validated on a larger cohort. This could be achieved by applying less restrictive inclusion criteria given the good tolerance of the procedure. In particular, patients with a FEV1 > 30% of predicted value could reasonably be included instead of a FEV1 > 50%.

The main limitations of the HF-NIV method are the current length of the procedure and the need for experts in ventilation technique. Nevertheless, these should be resolved by technical improvements of both MRI equipment as well as ventilation technique.

## Conclusion

UTE-HF-NIV is currently not ready to replace CT for lung nodule detection, despite performed at deep inspiration. Nonetheless, UTE-HF-NIV could be considered for growth evaluation follow-up in cases of assessable indeterminate nodules in a near future.

## Data Availability Statement

The raw data supporting the conclusions of this article will be made available by the authors, without undue reservation.

## Ethics Statement

The studies involving human participants were reviewed and approved by Vaud Ethics Committee (CER-VD, 2018-00438). The patients/participants provided their written informed consent to participate in this study.

## Author Contributions

ED: data curation, formal analysis, investigation, project administration, software, validation, visualization, writing—original draft, and writing—review and editing. MJ: formal analysis, investigation, and writing—review and editing. DR: investigation and writing—review and editing. SG: investigation. AC: investigation, resources, and writing—review and editing. JD: conceptualization, data curation, methodology, and writing—review and editing. JS and OL: methodology, investigation, and resources. FB: investigation and resources. J-BL: methodology, investigation, resources, and writing—review and editing. JP: conceptualization, formal analysis, funding acquisition, methodology, project administration, resources, supervision, validation, visualization, and writing—review and editing. AL: conceptualization, funding acquisition, investigation, methodology, resources, supervision, and writing—review and editing. CB-A: conceptualization, funding acquisition, investigation, methodology, project administration, resources, supervision, validation, visualization, writing—original draft, and writing—review and editing. All authors contributed to the article and approved the submitted version.

## Funding

This work was supported by grants from the Swiss National Science Foundation to CB-A and JP (n°320030-176241) and grants from the Leenaards Foundation, Switzerland (Bourse Relève 2017 Réf. 4890.0) to AL, Bourse Junior Clinical Scientist to DR.

## Conflict of Interest

The authors declare that the research was conducted in the absence of any commercial or financial relationships that could be construed as a potential conflict of interest.

## Publisher's Note

All claims expressed in this article are solely those of the authors and do not necessarily represent those of their affiliated organizations, or those of the publisher, the editors and the reviewers. Any product that may be evaluated in this article, or claim that may be made by its manufacturer, is not guaranteed or endorsed by the publisher.
